# Toxicological Effects of Copaiba Oil (*Copaifera* spp.) and Its Active Components

**DOI:** 10.3390/plants12051054

**Published:** 2023-02-27

**Authors:** Camila Castanho Cardinelli, Josiane Elizabeth Almeida e Silva, Rayssa Ribeiro, Valdir F. Veiga-Junior, Elisabete Pereira dos Santos, Zaida Maria Faria de Freitas

**Affiliations:** 1 Department of Drugs and Medicines, Faculty of Pharmacy, Federal University of Rio de Janeiro, Rio de Janeiro 21941-902, Brazil; 2 Department of Chemical Engineering, Military Institute of Engineering, Rio de Janeiro 22290-270, Brazil; 3 Department of Biological Sciences, Institute of Biological Sciences, Federal University of Amazonas, Manaus 69080-900, Brazil

**Keywords:** *Copaifera*, isolated compounds, toxicity, biological activity

## Abstract

Vegetable oils are among the most important traditional resources of Amazonia. Oleoresins are a type of oil that have interesting characteristics and highly bioactive properties with pharmacological potential. Oleoresins produced in the trunks of *Copaifera* (Fabaceae) spp. trees, known as copaiba oils, are made up of terpenes from the sesquiterpene (volatile) and diterpene (resinous) classes, but in amounts that vary between species and depending on several factors, such as soil type. Despite being used for medicinal purposes, via topical and oral application, the toxic effects of copaiba oils and their constituents are little known. The current paper reviews the toxicological studies, both in vitro and in vivo, described in the literature for copaiba oils, as well as the cytotoxic characteristics (against microorganisms and tumor cells) in in silico, in vitro and in vivo models for the sesquiterpenes and diterpenes that make up these oils.

## 1. Introduction

Oleoresins are natural mixtures of volatile/liquid and resinous terpenes, which together are heavy and solid at ambient temperature. Oleoresins present high viscosity and are liquid when just exudated from the trunks of trees, such as pines (*Pinus* spp.-Pinaceae), myrrh (Burseraceae), and copaibas (*Copaifera* spp.-Fabaceae). The oleoresin exudated from copaibas, known as copaiba oil, is obtained in amounts from 1 mL to 60 L. Its medicinal use was described as early as 1534 by the early European chroniclers during the invasion of the New World, when they observed its use by native populations for the treatment of wounds. Use of this oleoresin is now so widespread that copaiba is the most popularly known and exploited medicinal plant in Brazil. Copaiba oils can be administered as a local healing agent, as well as an anti-hemorrhoidal, purgative, anticarcinogenic, anti-inflammatory, antimicrobial, and anesthetic agent, though sufficient evidence of its efficacy and pharmacological safety are still required [1,2,3].

Scientific studies with copaiba oil seek to identify its constituents and correlate the isolated substances to the described biological effects. As for the chemical composition, the main constituents found in copaiba oils are sesquiterpenes (volatile fraction) and diterpenes (resinous fraction), which can vary qualitatively and quantitatively between species of the *Copaifera* genus and in relation to biotic and abiotic factors [4,5].

The volatile or sesquiterpene fraction can account for as little as 10–15% or up to 80% of the copaiba oil. Oils with fewer volatile constituents are more resinous, and, thus, are of little value on the market. On the other hand, oils rich in volatile sesquiterpenes, such as *α*-humulene and β-caryophyllene (BCP), are highly sought and are commercially valuable due to their uses in both the pharmaceutical and cosmetic industries. Biological activities attributed to BCP include insecticidal, antimicrobial, local anesthetic, anticancer, anti-inflammatory, and antileishmanial activity, while *α*-humulene has anti-inflammatory activities in commercial products, such as Acheflan^®^. Although bioactivities have been described for some of the sesquiterpenes isolated from copaiba oils, several biological properties are attributed to the intact oil only, where the substances act synergistically. As for the resinous, diterpenic fraction, the compounds most reported with regards to therapeutic activities are the kaurenoic, hardwickiic, polyaltic, and copalic acids, which have been attributed to anti-inflammatory, antiparasitic, antibacterial, antifungal, and antitumor activities [6].

**Figure 1:**
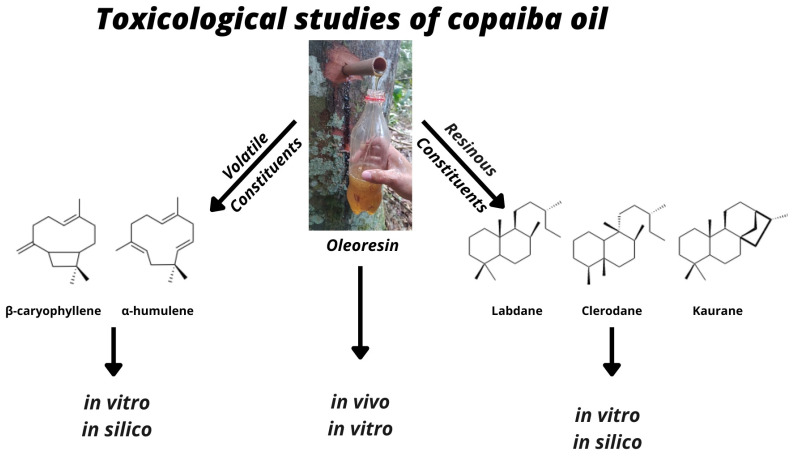
Toxicological studies of oleoresins and substances isolated from *Copaifera* spp.

However, studies are still needed to define the pharmacological properties of copaiba oils and their constituents in order to validate their safety for oral and topical use. For the copaiba oils, a number of studies have already been conducted to evaluate in vivo and in vitro cytotoxicity. However, there are fewer studies on the evaluation of their terpenic constituents (Figure 1).

Although some of the sesquiterpenes present in copaiba oils occur in other plant species and numerous in silico studies have been carried out, there are still few in vivo studies for these substances. For diterpenes, which are less common in other plant species, many of which are produced in a very specialized way in the *Copaifera* genus, in vitro studies predominate in the literature. The current study discusses the results of an extensive review of in vitro, in vivo, and in silico toxicity studies carried out for copaiba oils and their constituents, as a way of systematizing knowledge and allowing for the inference of toxic effects of some of these terpenes. Copaiba oils are often consumed on a large scale for medicinal purposes, and, thus, this study should highlight where investigations are still needed for their use to be considered safe.

## 2. Materials and Methods

The review of the toxicity of copaiba oils was carried out by retrieving studies published in different online databases, such as Google Scholar, PubMed, and Web of Science, using the following main search terms: “*Copaifera*”, “toxicity”, “copaiba diterpenes”, “copaiba sesquiterpenes”, “in vivo”, “in vitro”, and “in silico”.

## 3. Results

### 3.1. Cytotoxicity Assays Usually Performed in Natural Substances

With the increasing concern about the ethical aspects of performing in vivo experiments, assessment of toxicity by in vitro and especially in silico assays is preferred. However, when in vivo toxicity assays are performed, the main tests are based on OECD-423/2001, which establishes the median lethal dose (LD_50_), using the “Acute Class Toxicity” methodology for toxic dose testing with four dose levels (5, 50, 300, and 2000 mg/kg). LD_50_ is the dose of a given substance that kills 50% of a test population, usually measured in milligrams of substance per kilogram of test subjects’ body weight (mg/kg *bw*). Based on a review of the literature, tests have been performed on male Swiss albino mice, male Wistar rats, female rats, and albino rats, to assess hematological, histological, hepatotoxic, and biochemical parameters [7,8].

To analyze toxicity in vitro, the simplest test is performed using the nauplii of the *Artemia salina* microcrustacean, which act as a bioindicator, presenting a clear response to a reduced environmental component or in the presence of a specific toxic substance. The lethality of this organism has been used to identify biological reactions in which only a life or death output can occur. The most common in vitro test is the 3-(4,5-dimethylthiazol-2-yl)-2,5-diphenyltetrazolium bromide (MTT) assay, a colorimetric test that allows the number of living cells to be evaluated after their incubation with a toxic substance. A similar method is the 2,3-bis-(2-methoxy-4-nitro-5-sulfophenyl)-2H-tetrazolium-5-carboxanilide (XTT) assay, frequently used for the quantitative determination of cell proliferation and for determining the cytotoxic effects of substances. The neutral red (NR) test is also frequently used; this is based on the ability of cells with intact membranes to absorb and retain the neutral red dye in their lysosomes, the amount of dye being determined by absorbance at up to 540 nm. One simple method to detect maintained adherence of cells is the staining of attached cells with crystal violet (CV) dye, which binds to proteins and DNA. Cells that undergo cell death lose their adherence and are subsequently lost from the population of cells, resulting in a reduction in the amount of CV staining in a culture [9,10,11,12].

The most recent assays that have been developed are those that use computational tools to simulate different properties in biological media, the so-called in silico tests. To perform in silico toxicity tests, the main model currently used is online preADMET (http://preadmet.bmdrc.org/, accessed on 12 December 2022), which includes computational tools that evaluate the absorption, distribution, metabolism, excretion, and toxicity properties of a single substance. Other models used are the VEGA platform, version 1.1.5-b22, T.E.S.T software, version 4.2.1, and Toxtree, version 3.1.0, which assess intestinal toxicity, permeability, and absorption. Furthermore, the SwissADME platform (http://www.swissadme.ch/, accessed on 12 December 2022) evaluates physical-chemical, pharmacokinetic, and bioavailability properties, and the ADMET PredictorTM software (Simulation Plus, Lancaster, CA) presents potential toxicity parameters of a substance [13,14,15].

**Figure 2:**
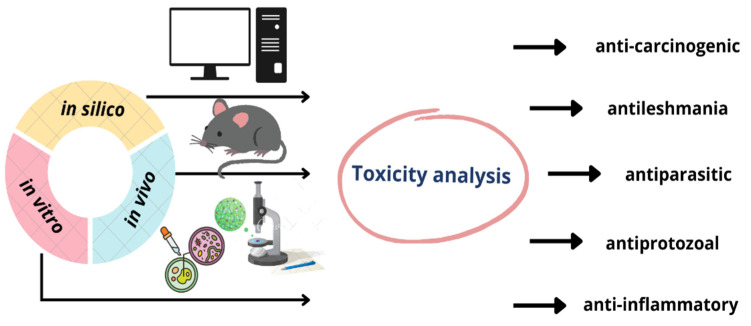
Biological activities that can result from in vivo, in vitro, and in silico toxicological studies.

The various substance assessment models used to evaluate toxicity are illustrated in Figure 2 with the types of biological activities that can also be indicated using such methods.

The literature review revealed that evaluation of cytotoxicity in human and tumor cells are among the most often analyzed with regards to the in vitro assays of copaiba oils and their constituents. Evaluation of the action against microorganisms is equally well studied, whether that be bacteria and fungi or protozoa such as *Trypanosoma cruzi* and *Plasmodium falciparum*, responsible for Chagas disease and malaria, respectively. Furthermore, assays that analyze physical-chemical, absorption, metabolism, and excretion properties of copaiba oils and their constituents also help understand several aspects, such as inflammatory processes.

### 3.2. In Vivo Toxicological Analyses of the Copaiba Oil and Its Constituents

In vivo studies are important to evaluate toxicity and indicate safety levels for the use of medicinal plants. In vivo toxicity evaluation studies were performed for copaiba oils extracted from different species of the *Copaifera* genus.

The acute toxicity of the copaiba oil from *Copaifera reticulata* Ducke was evaluated by oral administration (gavage) in female Wistar rats. The evaluation of acute oral toxicity was performed based on the OCDE-423/2001 guidelines, which determined the concentration of the doses and the number of animals per dose (three rats) used in the testing. An oleoresin solution diluted in 2% of Tween 80 was administered at 10 mL/kg. at an initial dose equivalent to 300 mg/kg up to 2000 mg/kg. One hour after administering the oleoresin doses, the behavioral, motor, and sensory functions were assessed to determine the neurotoxic effects in these rodents. There were no significant changes between the tested groups; in other words, the acute oral toxicity of this tested copaiba oil was found to be higher than the highest dose tested: 2000 mg/kg [7].

Studies on the fetotoxic effects of the copaiba oil from *C. reticulata* in a vaginal cream were evaluated in relation to the reproductive performance of albino Wistar rats. Females were randomly distributed into three groups, subjected to intravaginal treatment for 30 days and then mated with males in order to get pregnant. One control group received 0.5 mL of 0.9% sodium chloride, while another group was treated with the base vaginal cream. The test group received a 28.6 mg/kg dose of vaginal cream containing copaiba oil. The following evaluation criteria were used: maternal weight, maternal reproductive performance, fetal weight, placental weight, and placental index. The vaginal cream with 2.5% C. reticulata oleoresin did not show fetotoxic effects during the pre-implantation and organogenesis period [16].

A recent study evaluated treatment with the *C. reticulata* oleoresin in Wistar rats through the oral and intravaginal routes. In this study, the groups were treated with doses of 0.04 (oral route; 10 rats), 28 (intravaginal route; 5 rats), and 32 (intravaginal route; 5 rats) mg/kg/day. The control groups received 0.5 mL of distilled water (oral route; 10 rats) and 220 mg of base vaginal cream (vaginal route; 5 rats). Consequently, the subacute treatment with the *C. reticulata* oleoresin did not cause death or produce clinical signs of toxicity, including weight development, whether through the oral or the intravaginal route [17].

Oral toxicity was evaluated by administering a single high dose or lower repeated doses for 28 days of a commercial copaiba oil without botanical identification. Despite the known chemical differences between the copaiba oils from different *Copaifera* species, copaiba oils are commonly commercialized without botanical identification. In the acute toxicity test, a 2000 mg/kg dose of the oleoresin was administered via an intragastric tube (gavage) at 10 mL/kg. To evaluate the subacute toxicity, on the other hand, the animals received oral doses of 25, 50, and 100 mg/kg for 28 days. The control groups received 10% of Tween 80. There were no significant differences in the short-term toxicity tests of animals exposed to high oleoresin doses with absence of compromised tissue in the histological analysis [18].

Developmental toxicity was evaluated in pregnant Wistar rats that received a *C. reticulata* oleoresin emulsion in 2% of Tween 80. The volume administered in the intervention group was 10 mL/kg/day by oral gavage. There was a reduction in maternal food consumption at the beginning of pregnancy and a reduction in maternal weight gain at the two highest doses tested (1000 and 1250 mg/kg/day). These results indicated that the oleoresin proved to be toxic for the mother at oral doses equal to or higher than 1000 mg/kg/day. However, the oleoresin did not induce embryonic death at the doses tested. Overall, the 500 mg/kg/day dose proved to be both non-embryotoxic and safe for the mothers. Furthermore, there was no evidence of teratogenicity for any of the doses tested. Consequently, the estimated safe exposure level for humans, based on the non-observed adverse effect level (NOAEL) found in guinea pigs for maternal and developmental toxicity, was 500 mg/kg/day [8]. For females of reproductive age, the estimated safe dose for oral administration is 5 mg/kg/day [19].

The effects on reproductive performance following the oral administration of the *C. multijuga* oleoresin in male Wistar rats at the 200, 500, and 2500 mg/kg/day doses were evaluated during eight weeks, with no signs of toxicity. Treated rats were mated with untreated females and no stillborns or fetal malformations were found, which indicates absence of externally visible teratogenic effects. In summary, the results confirmed that oral treatment with this copaiba oil at the concentrations evaluated did not induce toxic effects on the male reproductive system, on the animals’ fertility, nor on the development of their offspring [20].

The clinical, histopathological, and toxicogenetic effects of eye drops containing 0.1% and 0.5% *C. multijuga* oleoresin were evaluated on alkali-induced superficial corneal ulcers in the left eyes of male Wistar rats. The oleoresin was diluted in 5% of Tween 80. There was an absence of toxicity on the eye surface for the two concentrations tests, and there were no signs of genotoxicity [21]. In another study using male Swiss albino mice for genotoxicity assessment, commercial copaiba oil at doses of 500, 1000, and 2000 mg/kg was administered by gavage. Doxorubicin and methyl methanesulfonate were used as positive controls. There was no increase in the size of the DNA tail in the use of the commercial oleoresin and its fractions, which demonstrates absence of genotoxicity [22].

Due to their anti-protozoal properties, copaiba oils are popularly used in the treatment of various endemic diseases. Leishmaniasis is one such disease that has high morbidity and mortality rates. Male and female BALB/c mice subcutaneously infected with *L. amazonensis* were treated with *C. martii* oleoresin formulations. The first formulation was a 4% cream for topical use, to which copaiba oil in Tween 80 (100 μg of copaiba oil for each gram of cream) was added. The second was an oral emulsion (100 mg/kg) and the third was a subcutaneous preparation of the copaiba oil in Tween and PBS (100 mg/kg). Glucantime^®^ was used as a control. Combined oral and topical treatments, and oral alone, caused significant reductions in lesion size. In addition to this, no behavioral or clinical changes were observed in the animals treated with the *C. martii* oleoresin, confirming that it is non-genotoxic [23].

Another endemic disease for which the action of copaiba oils has been studied is malaria, one of the most important parasitic diseases affecting inhabitants of tropical regions. The potential antimalarial effect of the *C. reticulata* oleoresin was evaluated in BALB/c mice infected with *Plasmodium berghei*, by using the oleoresin diluted in 4% of Tween 80. The blood analysis results indicated a reduction in parasitemia in all the treated animals, as well as presenting low in vivo toxicity to the animals. The oleoresin reverted the clinical manifestation of malaria in the infected animals, with improvement of biochemical and hematologic parameters, thus confirming the antimalarial effect [7].

The effects of copaiba oil on protecting the liver from damage induced by paracetamol were evaluated in 36 male Wistar rats. The animals were divided into groups, one group receiving a 0.63 mL/kg dose of copaiba oil from an unidentified species by gavage once a day for 7 days before the administration of paracetamol, while two other groups received 3.8 mL/kg either two hours before or two hours after receiving the paracetamol dose. The higher concentration of copaiba oil given just before the paracetamol did not present prophylactic effects; however, when used at a lower concentration for 7 days, it proved to be hepatoprotective in animals and did not significantly alter liver function, only showing a slight increase in bilirubin levels compared to the untreated control group and without any signs of cholestasis. Furthermore, the higher concentration given after the paracetamol also had therapeutic effects [24].

A further study from the same group evaluated the effect of a copaiba oil from an unidentified species on the liver damage induced by paracetamol compared to corn oil. Experimental groups of male Wistar rats were organized in a similar way to the prior study, wherein one group received 0.63 mL/kg of the oleoresin by gavage for 7 days before receiving the paracetamol dose, one received 3.8 mL/kg of oleoresin two hours before the paracetamol dose, and another received 3.8 mL/kg of oleoresin two hours after the paracetamol dose. The corn oil groups were set up like these but no effects on liver damage were observed for any these groups. The results also showed an hepatoprotective effect of the copaiba oil and demonstrated that there was no hepatic toxicity [25].

The evaluation of the hepatotoxicity of the *C. reticulata* oleoresin was performed through morphological and morphometric analyses in the liver of treated rats. Male Holtzman rats were induced to develop adjuvant arthritis to later be treated with 1.15 g/kg of the oleoresin via the oral route for 18 days. The arthritic animals treated with copaiba oil presented inflammatory foci, although fewer foci were seen when compared to their untreated counterparts. In rats treated with copaiba oil, however, the area of hepatocytes and their numbers were smaller than in untreated control rats, indicating warning signs for liver damage in relation to the use of this oleoresin [26].

Anti-inflammatory activity of a copaiba oil (*C. multijuga*) was evaluated in two in vivo models: mouse ear edema and rat paw edema. Hydrogel formulations containing copaiba oil nanoemulsions with positive and negative charges (PCN and NCN), by the presence and absence of cetyltrimethylammonium bromide, respectively, were generated. Both NCN and PCN inhibited the edema by 69 and 67% in the mouse ear model, and by 32 and 72% in the rat paw model, respectively. In both models, it was possible to visualize, by histological cuts, a decrease in epidermis hyperplasia, inflammatory cell infiltration, and vasodilation, demonstrating the anti-inflammatory activity of both the PCN and NCN treatments [27].

Alterations in the bladder-healing process of male Wister rats after the topical use of *C. reticulata* oleoresin were assessed. Rats were randomly divided into two groups: control group (CG) with 1 mL/kg of saline solution applied on the suture line, and the copaiba group (CpG), with 0.63 mL/kg of the copaiba oil applied on the suture line. Euthanasia was performed on the seventh day after surgery. Both groups showed adhesions to the bladder, with no statistically significant difference. The copaiba oil modified the healing process, improving the quantity of giant cells and vascular proliferation [28].

The effect of BCP, the major sesquiterpene of copaiba oil, was evaluated on systemic inflammation, oxidative status, and metabolism in rats with adjuvant-induced rheumatoid arthritis. This study also compared the actions of BCP with those reported by Ghizoni et al. [26], who evaluated the copaiba oil in arthritic rats. Healthy and arthritic Holtzman rats received 215 and 430 mg/kg of BCP orally once a day for 18 days. Both doses of BCP reduced the adjuvant-induced paw edema, swollen lymph nodes, and number of circulating and articular leukocytes. Furthermore, the 430 mg/kg dose abolished the increases in protein carbonyl groups and myeloperoxidase activity in the liver and plasma of arthritic rats, and, at both doses, the increased levels of reactive oxygen species and reduced glutathione in the arthritic liver were restored. These beneficial actions of the BCP were to the same extent as those reported for the *C. reticulata* oleoresin and, therefore, BCP is possibly responsible for the anti-inflammatory and antioxidant actions of the copaiba oil [29].

Kaurenoic acid from *C. officinalis* oleoresin and the oleoresin itself were evaluated for their antinociceptive effect, while the mechanism of action and possible adverse effects were also investigated in mice. The tail-flick test was carried out in male and female albino Swiss mice in order to test the antinociceptive effect of the oleoresin (10 mg/kg, intragastric) and kaurenoic acid (1 mg/kg). Intraplantar injection of capsaicin, allyl isothiocyanate (AITC), and complete Freund’s adjuvant (CFA) were used. The copaiba oil and kaurenoic acid had an antinociceptive effect in the tail-flick test in a dose-dependent manner, and this effect was reversed by naloxone (an opioid antagonist). Both *C. officinalis* oleoresin and kaurenoic acid reduced the nociception caused by capsaicin and AITC and produced an antiallodynic effect in the CFA model (after acute or repeated administration for 7 days) [30].

Menezes et al. [31] performed a systematic review aimed at defining the benefits related to the anti-inflammatory and healing capacity of copaiba-oil-based formulations on the oral cavity. Of the five studies reviewed, two reported beneficial wound healing effects, such as early reduction in the wound area and greater immature bone formation in the mandibles of the animal,; and two reported beneficial anti-inflammatory effects, such as reduced acute inflammatory reaction and more advanced tissue repair stage, including early formation of collagen fibrils with greater quantity, thickness, and better organization, as well as more expressive anti-inflammatory activity, and reduction in the edema intensity and the CD68 þ macrophage population.

### 3.3. In Vitro Toxicological Analyses of the Copaiba Oil and Its Constituents

#### 3.3.1. Cytotoxicity (Normal Cells)

Several in vitro studies have contributed to the understanding of the toxicity of copaiba oils. In order to evaluate the cytotoxicity of the *C. reticulata* oleoresin, peritoneal macrophages were infected with *Leishmania amazonensis* parasites and treated with copaiba oil. The cytotoxic effect of copaiba oil was evaluated in the J774G8 macrophage cell line (originally derived from the peritoneal exudate of BALB/c mice) by the sulforhodamine B assay. The selective index (SI) (ratio: CC_50_ J774G8 cells/IC_50_ protozoa) was calculated, where a value greater than 1.0 is considered more selective towards parasite killing. The results showed that the oleoresin was more toxic to *Leishmania* in both the promastigote and amastigote forms than to J774G8 macrophages [32]. Furthermore, it was assessed that, at the 500 mg/mL concentration, the *C. reticulata* oleoresin did not affect the viability of peritoneal macrophages [33].

The in vitro cytotoxic activity of *C. reticulata* oleoresin against GM 07492 human lung fibroblast cells was determined using the XTT assay. XTT reduction occurs extracellularly on the surface of the plasma membrane of live cells via transmembrane electron transport. This colorimetric reduction assay is often performed to assess the sensitivity of tumor cells to natural products and is widely used for the quantitative determination of cell proliferation. The copaiba oil concentrations used varied from 9.7 to 1250 μg/mL, dissolved in 0.2% of Tween 80. Concentrations of up to 39 μg/mL significantly reduced cell viability in relation to the negative controls (no treatment) [10].

The cytotoxic effect of free sodium diethyldithiocarbamate trihydrate (DETC) and nanoparticles incorporating DETC-beeswax and copaiba oil on peritoneal macrophages was assessed by the MTT assay. The treatments with 1 and 10 μM of free DETC showed a reduction in the viability of murine peritoneal macrophages. In contrast, the treatment with DETC-beeswax and copaiba oil nanoparticles did not affect cell viability. These results showed that incorporating DETC in beeswax and copaiba oil nanoparticles protects healthy cells, reducing the toxic effect of DETC [34].

A study assessing whether copaiba oil and one of its diterpenic constituents, kaurenoic acid, could increase the trypanosomicidal capacity of infected cells, evaluated the phagocytic index of peritoneal macrophages and cells of the human cervical adenocarcinoma cell lineage (HeLa). It was observed that, regardless of whether the cell was classed as phagocytic or non-phagocytic, both treatments promoted a significant reduction in the infection and proliferation rate of the amastigote forms of the parasite. Furthermore, the concentrations tested were not cytotoxic to the mouse peritoneal macrophages, with CC_50_ values of 46.25 μg/mL for copaiba oil and 70.63 μg/mL for kaurenoic acid [35].

Another study evaluated the cytotoxicity of a copaiba oil in 3T3-L1 mouse fibroblast cells by the MTT method at concentrations of 1, 10, 50, 100, 500, and 1000 µg/mL for 24 h. The results obtained showed that from 90% to 100% of the 3T3-L1 fibroblasts were still viable at the 100 µg/mL concentration. At 500 and 1000 µg/mL, however, there was a drop in viability to 70% and 73%, respectively [36].

The antiproliferative effect of one of the main sesquiterpenes present in copaiba oils, BCP, was tested in two normal cell lineages: 3T3-L1 (mouse fibroblast) and RGC-5 (human retinal ganglion cells). Using the MTT assay, the cells were treated with BCP at a concentration of 10 μM for 6 and 12 h. DMSO (0.1%) was used as a negative control and 5-fluorouracil (10 µM) as a positive control. The IC_50_ values obtained for the 3T3-L1 and RGC-5 cells were 133 µM and 98 µM, respectively. In addition to this, the compound presented low toxicity against these normal cells [37].

In a recent study, hardwickiic acid, a resinoid clerodane diterpene present in several copaiba oils, was evaluated for its cytotoxicity in a RAW 264.7 macrophage cell line by the resazurin assay, which measures cellular metabolic activity. Cells were treated in triplicate with a concentration of 1.01 to 632.71 μM of hardwickiic acid, in parallel with the negative control (no treatment), the positive control (curcumin), and the medium without cells as blank. The results showed a low CC_50_ value of 247.83 ± 6.32 μM for the hardwickiic acid against 29.99 ± 2.82 μM for curcumin, resulting in a selectivity index of SI of 7.85 [38].

The Alamar Blue technique was used to evaluate the cytotoxicity of six diterpenic acids commonly found in copaiba oils (kaurenoic, copalic, 3-hydroxy-copalic, kolavic-15-methyl ester, 3-acetoxy-copalic, and hardwickiic) against two cell lines (J774 murine macrophages and 3T3-L1 fibroblasts). The results showed that the substances evaluated at 5 mg/mL did not significantly reduce viability after 24 h of treatment in experiments with 3T3-L1 cells [39].

The antimicrobial potential of the *C. reticulata* oleoresin was evaluated against the causative agents of tooth decay and periodontitis via determination of the minimum inhibitory concentration (MIC), minimum bactericidal concentration (MBC), minimum inhibitory concentration of biofilm (MICB50), time-kill assay, and checkerboard assay. MIC and MBC values ranged from 6.25 to 200 mg/mL against the tested bacteria. The time-kill assay conducted with oleoresin concentrations between 50 and 100 mg/mL showed bactericidal activity against *Fusobacterium nucleatum* (ATCC 25586) and *Streptococcus mitis* (ATCC 49456) after 4 h, *Prevotella nigrescens* (ATCC 33563) after 6 h, *Porphyromonas gingivalis* (ATCC 33277) and *Lactobacillus casei* (clinical isolate) after 12 h, and *Streptococcus salivarius* (ATCC 25975) and *Streptococcus mutans* (ATCC 25175) after 18 h [10].

An emulsion of the *C. multijuga* oleoresin was evaluated in vitro against strains of *Streptococcus* spp. present in dental biofilm. The agar diffusion method of the oleoresin was used against strains of *Streptococcus mitis*, *S. constellatus*, and *S. salivarius* isolated from patients as well as standard strains of *S. mitis*, *S. mutans*, *S. sanguinis*, and *S. oralis*. A 1% chlorhexidine gel was used as a positive control and the base gel as the negative. The seeded plates were incubated at 37 °C for 12, 24, and 48 h. The experimental gel and the 1% chlorhexidine gel showed antibacterial activity against all the tested strains [40].

Diefenbach et al. [41] performed a systematic review to assess the antimicrobial action of several copaiba oils against oral pathogens compared to that of control substances. All studies showed that copaiba oil, regardless of its species, presented a bactericidal and/or bacteriostatic effect in the in vitro analyses. Only one study showed that the antimicrobial effect of the *C. officinalis* oleoresin was similar to that of chlorhexidine.

The antimicrobial action of a *C. langsdorffii* oleoresin paste and its components was assessed by the MIC in the agar gel technique against oral microorganisms that cause the formation of dentin bridge in dogs. However, calcium hydroxide P.A. alone had significantly greater antimicrobial action than the paste or the copaiba oil alone [42,43].

Nanostructured emulsions based on *C. langsdorffii* oleoresin, copaiba essential oil, and bullfrog (*Rana catesbeiana* Shaw) oil were investigated against fungi and bacteria related to skin diseases. The bioautography assay and the determination of MIC and antibiofilm activity were performed with species of the genera *Staphylococcus*, *Pseudomonas*, and *Candida*. The MIC assay, in association with the bioautography, revealed that some esters of palmitic and oleic acids (from bullfrog) together with copaiba essential oil constituents curcumene, himachalene, isothujol, and fenchene were likely responsible for the inhibition of some of the species. The nanostructured emulsions based on the copaiba oleoresin and essential oil improved the antimicrobial activity of the pure oils alone, especially against *Staphylococcus* and *Candida*, which are resistant to azoles. The bullfrog oil nanostructured emulsion showed a lower antimicrobial effect when compared to those of the copaiba oleoresin and essential oil [43,44].

In vitro cytotoxic, antioxidant, antibacterial, and antifungal assays and the time-kill curve studies of orange jessamine essential oil and BCP were carried out, as well as determination of the chemical composition of the essential oil. The cytotoxic activity of *Murraya paniculata* and BCP (7.8–500 μg/mL) was evaluated using the MTT assay on normal fibroblasts and hepatoma cells. The antioxidant activity was measured by the DPPH and ABTS assays. The MIC and time–kill curves (24 h) were performed against *Staphylococcus aureus*, *Escherichia coli*, *Salmonella typhimurium*, *Enterococcus faecalis*, *Aspergillus niger*, *A. fumigatus*, and *Fusarium solani* by the broth microdilution method. The essential oil showed only weak free radical scavenging capacity; however, it exhibited potential bactericidal and fungicidal activity for the microorganisms tested. In addition, the essential oil showed good selective cytotoxic activity against the hepatoma cells. BCP, the major compound of this essential oil, showed significantly lower results than the essential oil in all analyses, indicating that the in vitro biological activities of the essential oil may be due to the synergistic effect of the components of the oil [43,45].

Ferro et al. [46] performed a meta-analysis on the copaiba oil, its functions in metabolism and its properties as an anti-inflammatory agent, as assessed by in vitro and in vivo studies. The results presented in this meta-analysis demonstrated that the copaiba oil is an interesting alternative for treatment of pathologies such as chronic inflammation, infectious diseases, various types of cancer, autoimmune diseases, and as a vehicle for the absorption of other drugs.

#### 3.3.2. Cytotoxicity (Cancer Cells)

Table 1 describes the cytotoxic properties of the chemical constituents found in the copaiba oil against cancer cells. The *C. multijuga* oleoresin was evaluated against a B16F10 murine melanoma cell lineage by the MTT method. The results showed a cytotoxic action against B16F10 cells, with an IC_50_ of 457 mg/mL, drastically reducing the number of viable cells [47].

In one study, the oleoresin from a *Copaifera* sp. was evaluated against a murine melanoma tumor cell lineage (B16F10), a human breast adenocarcinoma (MCF-7), a human cervical adenocarcinoma (HeLa), a human hepatocellular carcinoma (HepG2), and human glioblastoma (MO59J, U343 and U251) lineages. Oleoresin at concentrations from 15 to 7630 µg/mL was tested, while solvent (Tween 80 at 0.25% in the culture medium) was used as a negative control and doxorubicin as a positive control. The results obtained by the XTT assay showed that the oleoresin was only active against the MCF7 cancer cells (IC_50_ = 488.90 μg/mL). In addition to this, copalic acid (isolated chemical constituent) was evaluated under the same experimental conditions, resulting in cytotoxic action on the HeLa and MO59J cells with IC_50_ values of 68.3 and 44.0 g/mL, respectively [9].

The compounds present in the *C. paupera* oleoresin were diluted in DMSO-methanol (1:9) and analyzed against the strains of murine lymphoma P-38, human lung carcinoma A549, human colon carcinoma HT-29, and human melanoma MEL-28. DMSO and taxol were used as negative and positive controls, respectively. The results of trypan blue assay indicated that the methyl copalate was active against the P-388, A549, HT-29, and MEL-28 cells, presenting IC_50_ values of 2.5 g/mL, 5 g/mL, 5 g/mL, and 10 g/mL, respectively [48].

The diterpenic compounds, kaurenoic acid, copalic acid, 3-hydroxy-copalic acid, kolavic acid-15 methyl ester, 3-acetoxy-copalic acid, and hardwickiic acid were evaluated against a variety of tumor cell lines, each at a concentration of 20 M. The cell lineages used were AGP01 (gastric), HCT116 (colorectal), MCF-7 (breast cancer), NIH-OVCAR (ovary), SKAMELL-4 (melanoma), and SF295 (human glioblastoma). The kolavic-15-methyl ester, kaurenoic, copalic, 3-hydroxy-copal, and 3-acetoxy-copalic acids presented little cytotoxicity in the AGP01, HCT116, and NIH-OVCAR cells. However, kaurenoic, 3-hydroxy-copal and 3-acetoxy-copalic acids showed cytotoxicity of nearly 20% in the SF295 line [39].

The MTT assay was used to evaluate the effect of BCP on BS-241 cells (mouse lymphoma cell lineage T-cells) and MoFir cells (human B-lymphocytes transformed with Epstein–Barr virus). BCP-activated caspase 3 has been observed to cause fragmentation of internucleosomal DNA, which is one of the main features of apoptosis. BCP showed concentration-dependent inhibition of proliferation and induced 85%–90% cell death in both cell lineages at concentrations of 4.8 μM and 4 μM, respectively [49]. Similar changes were observed in HCT116 colorectal cancer cells treated with BCP [47].

The evaluation of the inhibitory effect of BCP on the proliferation of tumor cell lineages was analyzed using the MTT assay for concentrations of 10, 20, 40, and 60 µM. The results demonstrated significant antiproliferative activity against HCT116 (colon cancer, IC_50_ = 19 µM), PANC-1 (pancreatic cancer, IC_50_ = 27 µM), and HT-29 (colon cancer, IC_50_ = 63 µM) cell lines, while exhibiting moderate or weak cytotoxic effects against ME-180 (cervical), PC-3 (prostate), K-562 (leukemia), and MCF-7 (breast) cancer cell lines. The results were compared with the respective reference standard drugs, tamoxifen, betulinic acid, and 5-fluorouracil [37].

BCP can be used to treat a wide range of cancer cells found in organ and muscle tissues, such as melanomas, hepatic cholangiocarcinoma, ductal carcinoma, and cholangiocarcinoma [50,51], owing to its synergistic impact with chemotherapeutic drugs such as sorafenib and doxorubicin. The chemosensitivity of cancer cells caused by BCP results in a reduction in the amount of chemotherapy administered. This combined activity has, thus, contributed to reduced treatment costs, fewer side effects, and less damage to the patients’ organs (heart, kidneys, brain, and liver) [52,53]. In addition to this, BCP serves as a cytotoxic agent inducing cell cycle arrest, apoptosis, and inflammation through production of reactive oxygen species (ROS). It also serves as a glioblastoma suppressor, allowing paclitaxel to diffuse across the membrane more easily, thus enhancing the anticancer effects of chemotherapeutic drugs [54].

**Table 1:** Cytotoxic properties of the chemical constituents of oleoresins from *Copaifera* spp.

*Copaifera* spp.	Material	Cytotoxic Activity	Reference
*C. paupera*	Methyl copalate	IC_50_ = 2.5 g/mL (P-388–murine lymphoma), IC_50_ = 5 g/mL (A549–human lung carcinoma), IC_50_ = 5 g/mL (HT-29–human colon carcinoma); IC_50_ = 10 g/mL (MEL-28–human melanoma).	[48]
*Copaifera* sp.	Kaurenoic acid	IC_50_ = 84.2 μM (MCF-7–human breast tumor, 45% growth inhibition) and IC_50_ = 44.7 μM (HCT8–human colon tumor, 45% growth inhibition).	[55]
*C. multijuga* Hayne	Oleoresin	IC_50_ = 457 μg/mL (B16F10–murine melanoma).	[47]
-	α-humulene	IC_50_ = 55 to 73 μM (MCF-7–human breast tumor), (PC-3–prostate tumor), (A549–lung tumor), (DLD-1–colorectal adenocarcinoma), (M4BEU–melanoma) and (CT-26–fibroblast).	[56]
-	β-caryophyllene oxide	IC_50_ = 3.95 µM (HepG2–hepatocyte carcinoma); IC_50_ = 12.6 µM (AGS–gastric adenocarcinoma); IC_50_ = 13.55 µM (HeLa–human cervical adenocarcinoma); IC_50_ = 16, 79 µM (SNU-1–gastric carcinoma); IC_50_ = 27.39 µM (SNU-16–gastric carcinoma).	[57]
*C. multijuga* Hayne	Kaurenoic, copalic, 3-hydroxy-copalic, 3-acetoxy-copalic and hardwickiic acids and kolavenic acid methyl ester	IC_50_ = 20 μM (AGP01–human gastric cancer), with 13% growth inhibition; IC_50_ = 20 μM (SF-295–human glioblastoma), with 18% growth inhibition.	[39]
*Copaifera* sp.	Copalic acid	IC_50_ = 68.3 μg/mL (MO59J–human glioblastoma); IC_50_ = 44.0 μg/mL (HeLa–human cervical adenocarcinoma).	[9]
-	β-caryophyllene oxide	IC_50_ = 8.94 × 10^−3^ mg/mL (A-2780–human ovarian cancer cell lineage).	[58]
-	β-caryophyllene oxide, α-humulene, trans-nerolidol and valencene	β-caryophyllene oxide with IC_50_ = 57.7 μg/mL, α-humulene with IC_50_ = 24.1 μg/mL, valencene with IC_50_ = 38.1 μg/mL and trans-nerolide with IC_50_ = 28.7 μg/mL (CaCo-2–human colorectal adenocarcinoma).	[59]
-	β-caryophyllene oxide; β-caryophyllene	IC_50_ = 28 µg/mL (HCT116–human colon tumor); IC_50_ = 32 µg/mL (PANC-1–pancreatic carcinoma); IC_50_ = 79 µg/mL (PC-3–prostate tumor); IC_50_ = 110 µg/mL (MCF-7–human breast tumor).	[37]

When measuring its effects on the signaling pathways of the SH-SY5Y neuroblastoma cell lineage, copaiba oil was found to activate the apoptosis signaling pathway and reduce viability of SH-SY5Y cells with an EC_50_ of approximately 400 mg/mL. In addition to this, BCP was able to negatively regulate the pI3K/Akt/mTOR signaling pathway. Consequently, copaiba oil regulates the signaling pathways associated with cell metabolism, growth, immunity, and apoptosis [60].

Another sesquiterpene of copaiba oils that showed cytotoxic activities against various types of cancer cells was caryophyllene oxide. Its cytotoxic effect was observed in cancer cell lineages of HeLa (human cervical adenocarcinoma cells), HepG2 (human leukemia cancer cells), AGS (gastric adenocarcinoma), SNU-1 (human gastric cancer cells), and SNU-16 (human cancer cells stomach cancer cells). The results showed that cell growth inhibition, assessed by the MTT assay, was dose-dependent and cell-specific. Caryophyllene oxide was more potent against HepG2, AGS, HeLa, SNU-1, and SNU-16 cells, with IC_50_ values of 3.95, 12.6, 13.55, 16.79, and 27.39 µM, respectively [57].

The effects of caryophyllene oxide on the PI3K/AKT/mTOR/S6K1 and MAPK signaling pathways in human prostate (PC-3) and breast (MCF-7) cancer cell lines was found to increase generation of ROS from mitochondria, which induced apoptosis (assessed by annexin V and TUNEL staining), loss of mitochondrial membrane potential, release of cytochrome c, activation of caspase-3, and cleavage of PARP, as well as affecting the expression of several gene products that mediate cell proliferation and metastasis. Consequently, the use of an inhibitor of ROS generation significantly inhibited caryophyllene-oxide-induced apoptosis [61].

The antiproliferative effect of five sesquiterpenes (BCP, α-humulene, valencene, caryophyllene oxide, and trans-nerolidol) was evaluated by means of the MTT assay. Human colon adenocarcinoma (CaCo-2) cells were incubated with individual sesquiterpenes at concentrations of 0–50 µg/mL (diluted in DMSO) for 72 h, while doxorubicin was used as a positive control. All sesquiterpenes (with the exception of BCP) inhibited the proliferation of these cancer cells in a concentration-dependent manner, with humulene and nerolidol as the most effective [59].

On the other hand, humulene isomers in combined treatment with BCP were reported to be more effective in reducing the proliferation of cells of the MCF-7, DLD-1, and L-929 lines than when used separately. α-Humulene alone (32 µg/mL) inhibited cell growth by nearly 50%, but, combined with 10 µg/mL of BCP, there was 75% inhibition. In addition to this, BCP (at 10 µg/mL) potentiated the anticancer activity of paclitaxel when used in combination for the treatment of DLD-1 cells, increasing paclitaxel activity nearly 10 times. It is possible that BCP increases drug accumulation through a different mechanism of action, as it caused intracellular accumulation of calcein, but not of verapamil, an inhibitor of P-glycoprotein and multidrug-linked protein transporters. These findings imply that BCP helps the passage of paclitaxel across the membrane, amplifying its anticancer action [56].

Polyaltic acid was reported to have no cytotoxicity against two tumor cell lineages (HeLa and MCF-7) and the normal MCF-10A cell lineage. In this same study, cytotoxicity analyses were performed, in which kaurenoic acid showed efficacy with IC_50_ values from 50.2 to 65.2 μg/mL against A-375, HepG2, HT-29, HCT116 and MB-231 cell lines [62].

To evaluate the cytotoxicity of copalic acid and kaurenoic acid against cells of the human colon adenocarcinoma cell lineage (CaCo-2), the sodium resazurin (7-hydroxy-3H-phenoxazin-3-one-10-oxide) assay was used. The acids were tested at concentrations ranging from 3.91 to 500 µM (3.91; 7.81; 15.62; 31.25; 62.5; 125; 250; and 500 µM). The IC_50_ values for copalic and kaurenoic acids in CaCo-2 cells were 237.4 and 154.0 μM, respectively [63].

When examining the cytotoxic effects of clerodane diterpenes, including kolavic acid, good results were observed against cells of the cancer cell lineages, A549 (lung carcinoma-IC_50_ = 2.97 mg/mL), MCF-7 (breast cancer-IC_50_ = 2.15 mg/mL), and HT-29 (colon cancer-IC_50_ = 1.91 mg/mL) [64]. Another study evaluated the same compounds, but using human cervical carcinoma (HeLa) cells, in which the authors obtained an IC_50_ of 2.6 mg/mL, showing that clerodane diterpenes are potentially active against these cancer cell lines [65].

The cytotoxicity effects of clerodane diterpenes at several concentrations were tested against ovarian carcinoma (PA-1), breast adenocarcinoma (MCF-7), oral cell carcinoma (KB), and cervical carcinoma (C33A) cell lines, which presented IC_50_ values of 13.415, 28.001, 29.778, 14.333, mg/mL, respectively, as assessed by the MTT assay. Consequently, the compounds tested proved to be active against all the cancer cell lines tested [66].

In an investigation carried out with kaurenoic acid at a concentration of 78 μM, it was found that there was 95% inhibition of the growth of CEM leukemic cells and 45% inhibition of MCF-7 and HCT8 cancer cells. However, methyl copalate was active against cells of the P-388 (DBA/2 mouse lymphoid neoplasm-IC_50_ of 2.5 μg/mL), A549 (human lung carcinoma-IC_50_ of 5.0 μg/mL), HT-29 (human colon carcinoma-IC_50_ of 5.0 µg/mL), and MEL-28 (human melanoma-IC_50_ of 10.0 µg/mL) lineages, which kaurenoic was not [55].

Methyl copalate is a promising molecule when it comes to the in vitro results against cancer cell lines, as potent cytotoxic substances are considered when the IC_50_ is lower than 10 M. The copaiba oleoresins, including several isolated substances, have demonstrated anticancer effects. In the general context of isolated substances, it is understood some may have cytotoxic effects on certain tumor cell lines and not others; however, there may be synergism of the diterpene and sesquiterpene substances when combined or in the oleoresin *in natura*, justifying their biological effects [4].

#### 3.3.3. In Silico Toxicological Analyses of the Copaiba Oil and Its Constituents

To evaluate the efficacy of a bioactive compound, it should be stable and act on the target at a suitable concentration. The toxicity analysis of new compounds is an essential component of the characterization process for a new medication. Experimental animal models are labor-intensive and often time-consuming. Therefore, virtual screening using bioinformatics in the search for compounds that have biological activity should be performed mainly in initial prospecting studies, as a starting point for future analyses. The absorption, distribution, metabolism, excretion, and toxicity (ADMET) evaluation determines the potential properties of the compounds to be studied and their intoxication mechanisms. With virtual study, predictions on the physical-chemical, pharmacokinetic, and bioavailability properties of the compounds are observed [15,67].

Caryophyllene oxide was analyzed using the VEGA, T.E.S.T., and Toxtree platforms for the prediction of toxicity. This compound presented cutaneous permeability and low intestinal absorption and was considered as having high potential for toxic effects [13]. In another study, its toxicological and mutagenic effects (based on the Ames test), via the preADMET online server (http://preadmet.bmdrc.org/, accessed on 12 December 2022), showed mutagenicity generated by their epoxide group, a highly reactive and toxic functional group. Predictions of the carcinogenic effect are evaluated according to data from the National Toxicology Program (NTP) and the USA/FDA, which are predefined data from in vivo studies carried out in rats and mice for 2 years. The analysis of the potential for intestinal absorption and passive transport of drugs with fractions absorbed as a function of time can also be evaluated; caryophyllene oxide presented a mean permeability value of 56.3475 nm/s. In addition to this, regarding its penetration into the blood–brain barrier, it presented a value of 3.7524, which could indicate potential side-effects, as compounds that have penetration values greater than 1 are classified as active and can be toxic to the central nervous system [14,68].

Humulene was evaluated with the VEGA platforms, version 1.1.5-b22, the T.E.S.T. software, version 4.2.1, and Toxtree, version 3.1.0, showing carcinogenic potential [13]. Its α-humulene isomer showed carcinogenic potential, by analysis with the preADMET software. Other compounds of the sesquiterpene class were evaluated for mutagenic effects by means of the Ames test (preADMET), such as α-copaene, α-cubebene, and trans-α-bergamotene, showing no mutagenicity. As for the BCP, δ-cadinene, β-elemene, α-selinene, and β-bisabolene compounds, mutagenicity was evidenced [14].

Kaurenoic acid was evaluated for physical-chemical, pharmacokinetic, and bioavailability properties using the SwissADME platform (http://www.swissadme.ch/, accessed on 12 December 2022), and its chemical structure was drawn in a molecular sketcher interface and converted to SMILES notation. Among the analyses performed on this diterpene, its moderate solubility in water and high gastrointestinal absorption stood out [15]. In another study, the ADMET PredictorTM software (Simulation Plus, Lancaster, CA, USA) was used to assess the potential for toxicity where kaurenoic acid presented acute toxicity in rats [69].

## 4. Conclusions

Despite the numerous studies on oleoresins from the *Copaifera* genus, as well as its constituents, there are still many important gaps to be addressed in other fields. In the current study, it was concluded that there is a need to conduct new research on terpenes separately, in order to improve knowledge about their isolated actions. In addition, there is a need for new, more robust in vivo toxicological studies to ensure the safe dose of copaiba oleoresin use, mainly due to the lack of clinical studies in humans.

## Data Availability

Not applicable.
